# Supplementary material for: Functional Recovery by Transplantation of Human iPSC-Derived A2B5 Positive Neural Progenitor Cell After Spinal Cord Injury in Mice
Source: Int J Mol Sci. 2025 Sep 13;26(18):8940. doi: 10.3390/ijms26188940 (PMC12469384; doi:10.3390/ijms26188940)
Supplement: Supplementary file 1 [file ijms-26-08940-s001.zip › Supplementary Figure S1.pdf]

Supplementary Figure S1

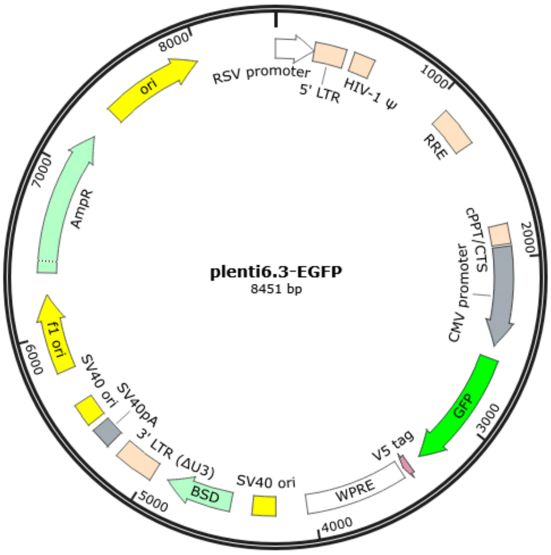

Supplementary Figure S1. Vector map for pLenti6.3 lentiviral vector encoding enhanced green fluorescent protein (EGFP) driven by the constitutive CMV promoter.
